# Unveiling the Pernicious Truth: A Case Report on the Rare Presentation of Severe Vitamin B12 Deficiency

**DOI:** 10.7759/cureus.101860

**Published:** 2026-01-19

**Authors:** Olawale O Akinola, Anvitha Mandapati, Nathan Douthit

**Affiliations:** 1 Internal Medicine, East Alabama Medical Center, Opelika, USA; 2 Internal Medicine, Edward Via College of Osteopathic Medicine, Auburn, USA

**Keywords:** clinical case, hemolytic anaemia, intramedullary hemolysis, pernicious-anemia, vitamin b 12 deficiency

## Abstract

Vitamin B₁₂ deficiency typically causes megaloblastic anemia but can rarely present with hemolytic features, leading to diagnostic confusion with autoimmune or microangiopathic processes. Pernicious anemia, an autoimmune cause of severe B₁₂ deficiency, is frequently underrecognized and may coexist with other autoimmune disorders.

We report a 45-year-old woman with Hashimoto’s thyroiditis who presented with dizziness, profound fatigue, and symptomatic anemia. Laboratory evaluation revealed severe macrocytic anemia (hemoglobin 3.5 g/dL, mean corpuscular volume (MCV) 136.9 fL) with thrombocytopenia, elevated lactate dehydrogenase (LDH) (2905 U/L), indirect hyperbilirubinemia, and suppressed haptoglobin, findings suggestive of hemolysis. Peripheral smear showed macrocytosis and marked anisopoikilocytosis without schistocytes. Coombs testing was negative, and G6PD levels were normal. Vitamin B₁₂ was severely reduced (73 pg/mL), and anti-intrinsic factor antibodies were positive, confirming pernicious anemia presenting as hemolytic anemia. Intensive intramuscular cyanocobalamin therapy led to rapid hematologic recovery, including normalization of platelet count within two weeks and improvement of hemoglobin to 8.4 g/dL.

Pernicious anemia can mimic hemolytic anemia, risking misdiagnosis as autoimmune hemolysis or thrombotic microangiopathy. Negative Coombs test, mild reticulocytosis relative to anemia severity, and macrocytosis provide critical diagnostic clues. Awareness of autoimmune clustering such as Hashimoto’s thyroiditis with pernicious anemia can prompt timely recognition. Early vitamin B₁₂ replacement reverses hematologic abnormalities and prevents irreversible neurologic damage.

## Introduction

Anemia is defined as a reduction in red blood cell mass, hemoglobin concentration, or hematocrit, resulting in impaired oxygen delivery to tissues. The World Health Organization (WHO) estimates that anemia affects around 500 million women of reproductive age and 269 million children worldwide [1]. In 2019, anemia was reported in 30% of non-pregnant and 37% of pregnant women aged 15-49 years [2]. According to WHO criteria, anemia is diagnosed when hemoglobin levels fall below 13 g/dL in men and below 12 g/dL in nonpregnant women [3]. Anemia is multifactorial and can be classified by red blood cell size (microcytic, normocytic, or macrocytic), onset (acute or chronic), etiology (nutritional deficiency, hemolysis, marrow failure, chronic inflammation, or inherited disorders), and severity (mild, moderate, or severe) [4,5]. Among global causes, nutritional deficiencies, particularly iron deficiency, remain predominant [2]. However, vitamin B₁₂ deficiency is a clinically significant but often overlooked cause, typically producing megaloblastic anemia due to defective DNA synthesis and ineffective erythropoiesis [5]. Efficient absorption of vitamin B₁₂ requires gastric intrinsic factor and formation of a B₁₂-intrinsic factor complex [6]. Severe deficiency caused by gastric bypass surgery, malabsorption, or autoimmune destruction of intrinsic factor (pernicious anemia) usually results in megaloblastic anemia but can, rarely, present with hemolytic anemia, as described in this case. Pernicious anemia is an autoimmune disorder characterized by antibodies against intrinsic factor or gastric parietal cells, impairing vitamin B₁₂ absorption [6]. It affects roughly 0.1% of the general population and up to 1.9% of those over 60 years, accounting for 20-50% of adult B₁₂ deficiency cases [7]. Historically, it was a fatal illness with patients typically surviving only one to three years after diagnosis until the discovery in the 1920s that liver ingestion improved survival and, subsequently, that vitamin B₁₂ was the curative factor [8]. These discoveries earned Minot and Murphy the Nobel Prize in 1934. With prompt recognition and lifelong supplementation, the prognosis is now excellent, though the condition remains underdiagnosed [6]. Definitive diagnosis depends on detecting parietal cell or intrinsic factor antibodies, and delayed treatment can lead to irreversible neurologic sequelae such as subacute combined degeneration of the spinal cord [9].

## Case presentation

A 45-year-old woman with a history of combined systolic and diastolic heart failure (automated implantable cardiac defibrillator in situ), essential hypertension, and hypothyroidism secondary to Hashimoto’s thyroiditis presented with dizziness and lightheadedness occurring during dinner. She reported nausea and transient blurring of vision without syncope or trauma. Over the preceding two weeks, she experienced increasing fatigue, which she attributed to her thyroid disease. She denied chest pain, palpitations, dyspnea, or seizures. No gastrointestinal or urinary symptoms were reported, though she noted intermenstrual bleeding for three months. Her medications included metoprolol, bumetanide, amiodarone, levothyroxine, and digoxin. On examination, she appeared pale and somnolent. Vitals were blood pressure of 109/62 mmHg, heart rate of 118 bpm, respiratory rate of 20/min, SpO₂ 100% (room air), and temperature 98.7°F. There was no cyanosis or jaundice. Cardiopulmonary, abdominal, and neurological examinations were unremarkable. Laboratory results (Table 1) revealed severe macrocytic anemia (hemoglobin 3.5 g/dL, mean corpuscular volume (MCV) 136.9 fL) with thrombocytopenia (56 × 10⁹/L). Hemolysis markers were deranged: lactate dehydrogenase (LDH) 2905 U/L, indirect bilirubin 1.6 mg/dL, and haptoglobin < 30 mg/dL. Iron and ferritin were normal, and thyroid testing confirmed uncontrolled hypothyroidism (thyroid-stimulating hormone (TSH) 11.2 µIU/mL). Serum vitamin B₁₂ was profoundly low (73 pg/mL).

**Table 1:** Initial Laboratory Investigations TSH: thyroid-stimulating hormone, LDH: lactate dehydrogenase

Test	Result	Reference Range	Interpretation
Hematology			
Red blood cell count	0.79 × 10^12/L	3.4 – 5.0 × 10^12/L	↓ Severe reduction
Hemoglobin	3.5 g/dL	11.7 – 15.5 g/dL	↓ Severe anemia
Hematocrit	0.108	36 – 46%	↓ Low
Mean corpuscular volume (MCV)	136.9 fL	80 – 100 fL	↑ Macrocytosis
Reticulocyte count	0.026	0.5 – 2.5%	↓When corrected
Platelets	56 × 10^9/L	150 – 450 × 10^9/L	↓ Thrombocytopenia
White blood cells	4.5 x 10^9/L	4.0 – 11.0 × 10^9/L	Normal
Hemolysis Markers			
Total bilirubin	2.4 mg/dL	0.3 – 1.1 mg/dL	↑ Elevated
Indirect bilirubin	1.6 mg/dL	<1.0 mg/dL	↑ Elevated
LDH	2905 U/L	98 – 192 U/L	↑ Markedly elevated
Haptoglobin	<30 mg/dL	44 – 215 mg/dL	↓ Reduced
Iron Studies			
Serum iron	256 µg/dL	28 – 170 µg/dL	↑ Elevated
Transferrin saturation	0.92	18 – 48%	↑ Elevated
Ferritin	192.9	12 – 300 ng/mL	Within range
Vitamin / Endocrine			
Vitamin B12	73 pg/mL	180 – 914 pg/mL	↓ Severe deficiency
Folate	12 ng/mL	3 – 17 ng/mL	Normal
TSH	11.2 µIU/mL	0.34 – 5.6 µIU/mL	↑ Elevated
Free T4	0.94 ng/dL	0.7 – 1.85 ng/dL	Normal
Other Tests			
HIV serology	Negative	—	Negative
Hepatitis panel	Negative	—	Negative

**Figure 1:**
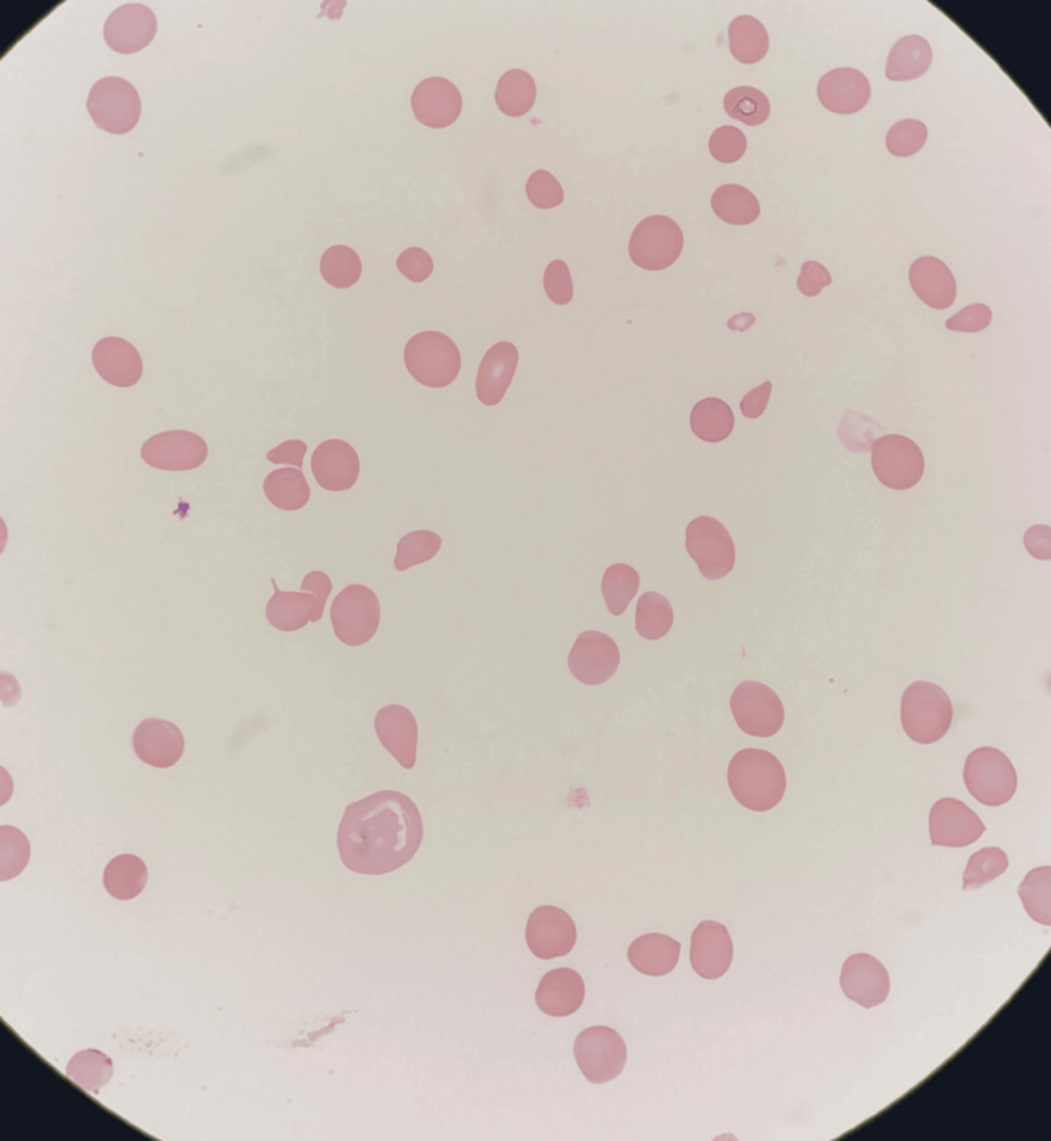
Peripheral Blood Smear

CT of the abdomen revealed no mass lesions, and pelvic ultrasound identified a benign nabothian cyst. Peripheral smear (Figure 1) demonstrated macrocytosis, anisopoikilocytosis, ovalocytes, elliptocytes, teardrop cells, and nucleated red cells, without schistocytes.

She was transfused with three units of packed red cells and started on intramuscular cyanocobalamin 1000 μg daily for seven days, then weekly for four weeks, and monthly thereafter. Rapid hematologic improvement followed (Table 2). G6PD level was normal, and a negative Coombs test excluded autoimmune hemolytic anemia. Anti-intrinsic factor antibodies were positive, confirming pernicious anemia as the etiology of severe B₁₂ deficiency with secondary hemolysis and thrombocytopenia.

**Table 2:** Hematologic Profile at Discharge and Two Weeks Post-discharge

Parameter	At Discharge	Two Weeks Post-Discharge	Interpretation / Trend
Red blood cell count	2.40 × 10^12/L	2.63 × 10^12/L	Improving, still low
Hemoglobin	8.3 g/dL	8.4 g/dL	Rising from nadir, remains anemic
Hematocrit	0.248	0.265	Improving
Mean corpuscular volume (MCV)	103.3 fL	100.7 fL	Trending toward normal
Platelet count	39 × 10^9/L	324 × 10^9/L	Recovered to normal
White blood cell count	4.3 × 10^9/L	7.6 × 10^9/L	Normal, improved

Clinical timeline

Over three months, the patient experienced progressive fatigue and exertional dyspnea, with dizziness worsening in the two weeks before presentation. Admission labs revealed severe macrocytic anemia (hemoglobin 3.5 g/dL, MCV 136.9 fL), thrombocytopenia, and biochemical evidence of hemolysis, with profoundly low vitamin B₁₂. She was promptly treated with packed red blood cell transfusions and intramuscular vitamin B₁₂, leading to rapid symptomatic improvement. By the third hospital day, the reticulocyte activity had improved. At discharge on day four, her hemoglobin had risen to 8.3 g/dL, and she transitioned to outpatient B₁₂ therapy. At her two-week follow-up, hemoglobin and macrocytosis continued to improve, and platelet counts normalized. Additionally, intrinsic factor antibodies confirmed the diagnosis of pernicious anemia. The patient reported feeling “dramatically better,” expressed full understanding of her diagnosis, and demonstrated clear commitment to lifelong B₁₂ replacement.

## Discussion

This case highlights a rare manifestation of vitamin B₁₂ deficiency presenting as hemolytic anemia rather than the classic megaloblastic form. The patient exhibited profound anemia (hemoglobin 3.5 g/dL), elevated LDH, indirect hyperbilirubinemia, and low haptoglobin, all consistent with hemolysis. Yet, the reticulocyte index (0.3) indicated ineffective erythropoiesis rather than brisk peripheral destruction.

On physical examination, the patient appeared pale and markedly fatigued, with sinus tachycardia reflecting the severity of her anemia, though she remained normotensive and clinically stable. There was no scleral icterus or cutaneous jaundice despite biochemical evidence of hemolysis, and no petechiae or purpura to suggest consumptive or microangiopathic processes. Cardiopulmonary examination revealed regular rhythm without murmurs and clear lung fields. The abdomen was soft and non-tender, with no hepatosplenomegaly. Neurologic examination showed preserved mental status, motor strength, sensation, reflexes, and coordination, indicating absence of overt neurologic sequelae of vitamin B₁₂ deficiency at presentation.

Differential diagnosis

Given her age and menstrual history, iron deficiency anemia was considered but excluded by normal iron studies and macrocytosis. Autoimmune hemolytic anemia (AIHA) was considered due to elevated LDH, indirect hyperbilirubinemia, and low haptoglobin, but excluded by a negative Coombs test. Microangiopathic hemolytic anemia (MAHA), including thrombotic thrombocytopenic purpura (TTP) and hemolytic uremic syndrome (HUS), was ruled out by the absence of schistocytes, neurologic deficits, or renal impairment. None of her medications are typical triggers for drug-induced hemolysis, and G6PD deficiency was excluded. Ultimately, the combination of macrocytosis, autoimmune background (Hashimoto’s thyroiditis), markedly low B₁₂, and positive anti-intrinsic factor antibodies confirmed pernicious anemia. This careful diagnostic approach prevented misclassification as AIHA or TTP, avoiding unnecessary corticosteroids or plasma exchange.

Pathophysiology of hemolysis in vitamin B₁₂ deficiency

B₁₂ deficiency disrupts DNA synthesis in erythroid precursors, creating fragile megaloblasts that undergo apoptosis within the marrow (intramedullary hemolysis) [5,6]. These cells release LDH and bilirubin, mimicking hemolysis but lacking an immune mechanism, hence the negative Coombs test [10].

Autoimmune context

Pernicious anemia commonly coexists with other autoimmune diseases [6,11]. Autoimmune thyroid disease, particularly Hashimoto’s thyroiditis, is observed in 10-40% of patients with autoimmune gastritis or pernicious anemia, underscoring shared immunologic mechanisms [11]. This overlap provided an important diagnostic clue in our patient.

Response to treatment

Following transfusion and cyanocobalamin therapy, the patient’s platelet count normalized within two weeks, and hemoglobin rose from 3.5 g/dL to 8.4 g/dL, confirming marrow recovery [7]. Lifelong B₁₂ supplementation was emphasized to prevent relapse and irreversible neurologic damage.

Limitations

This case illustrates a rare hemolytic presentation of pernicious anemia and provides well-documented diagnostic evidence and treatment response. However, as a single case, its generalizability is limited, and the absence of longer-term follow-up is an additional constraint.

## Conclusions

Vitamin B₁₂ deficiency, most often due to pernicious anemia, can occasionally manifest with hemolytic features instead of the classic megaloblastic presentation. In this case, the coexistence of Hashimoto’s thyroiditis provided a vital clue to an underlying autoimmune mechanism. Recognizing such atypical presentations prevents misclassification and avoids inappropriate therapy. Prompt diagnosis and lifelong vitamin B₁₂ supplementation can rapidly restore hematologic function and avert irreversible neurologic sequelae.
